# Effectiveness of natural biomaterials in the protection and healing of experimentally induced gastric mucosa Ulcer in rats

**DOI:** 10.1007/s11033-023-08776-9

**Published:** 2023-09-23

**Authors:** Doaa R.I. Abdel-Gawad, Marwa A. Ibrahim, Usama K. Moawad, Shaimaa Kamel, Hossny A. El-Banna, Ahmed H. El-Banna, Walid Hamdy Hassan, Fatma I. Abo El-Ela

**Affiliations:** 1 Lecturer of Toxicology and Forensic Medicine, Faculty of Veterinary Medicine, Beni-Suef University, Beni Suef, Egypt; 2 Department of Biochemistry and Molecular Biology, Faculty of Veterinary Medicine, Cairo University, Giza, 12211 Egypt; 3 Department of Histology and Cytology, Faculty of Veterinary Medicine, Beni-Suef University, Beni-Suef, Egypt; 4 Faculty of Veterinary Medicine, Cairo University, Giza, Egypt; 5 Michael Sayegh Faculty of Pharmacy, Aqaba University of Technology, Aqaba, Jordan; 6 Mycology and Immunology Department, Faculty of Veterinary Medicine, Beni-Suef University, Beni-Suef, 62511 Egypt; 7 Department of Pharmacology, Faculty of Veterinary Medicine, Beni-Suef University, 62511 Beni-Suef, Egypt

**Keywords:** Gastric ulcer, Propolis, Ginseng, Amygdalin, Histopathology, leukotriene4

## Abstract

**Background:**

A gastric ulcer is a painful lesion of the gastric mucosa that can be debilitating or even fatal. The effectiveness of several plant extracts in the therapy of this illness has been demonstrated in traditional pharmacopoeias. **Aim**: this study was aimed to see if propolis, ginseng in normal or nano form, and amygdalin might help in preventing the ulcerative effects of absolute ethanol.

**Methods:**

Gastroprotective properties of pretreatments before ethanol gavage in rats were compared to omeprazole. The ulcer and stomach parameters (ulcerated regions) were measured (mm^2^), ulcer inhibition percentage, the stomachs were assessed macroscopically with gastric biopsy histological examinations.

**Results:**

Amygdalin, normal and nano ginseng, nano propolis followed by propolis all showed great efficacy in protecting the cyto-architecture and function of the gastric mucosa. The number of ulcerated sites was greatly reduced, and the percentage of stomach protection was increased. Histopathological examination had confirmed great protective effects of the nanoformulations followed by amygdalin. The protection and healing rate was completed to about 100% in all tested materials while ulcer areas were still partially unhealed in normal propolis and omeprazole. Quantitative assay of the m-RNA levels Enothelin 1(ET-1), leukotriene4 (LT-4), and caspase 3(Cas-3) genes and Histamine were done and revealed significant up-regulations in ethanol group and the maximum protective effect was reported with ginseng nano, moreover the histamine content was significantly decreased with nano- formulated extracts.

**Conclusion:**

Amygdalin and the nanoformulated ginseng and propolis had exhibited a marked protective effect against the ulcerative toxic effects of ethanol.

## Introduction

A gastric ulcer is representing one of the most common gastroduodenal illnesses in humans and animals. It may be debilitating or life-threatening if the stomach is ruptured and followed by haemorrhage [1]. The major causes of peptic ulcers include an imbalance in gastric hydrochloric acid and pepsin production, as well as a loss in the cytoprotective capabilities of the gastroduodenal mucosal barrier [2]. In recent years, it has been widely accepted that *Helicobacter pylori* (HP) infection is the primary cause of peptic ulcers [3]. Other variables, such as nonsteroidal anti-inflammatory medicines (NSAIDs) like acetylsalicylic acid (ASA), fundamentally persistent emotional stress, overconsumption of alcohol and related beverages, and smoking of tobacco, are well documented to have a role in gastric ulcer formation [4].

Ethanol penetrates the gastrointestinal mucosa quickly, resulting in membrane damage, cell exfoliation, erosion, and ulcer development. Ethanol-induced gastric ulcer models are extensively used to investigate the development and treatment of ulcerative colitis in humans [5].

The ethanol-induced ulcer model in rats or mice is still one of the most commonly used methods in the study of active compounds’ therapeutic and/or preventative actions, particularly those derived from plant extracts. Indeed, ethanol has been linked to significant stomach injury and duodenal mucosa by causing disturbances in the environmental equilibrium of gastroduodenal cavities [6].

Traditional peptic ulcer treatments focus on reducing the causes of gastric, like proton pump inhibitors (PPIs), histaminic H_2_-receptor antagonists, anticholinergics, and antacids for enhancing the protection level of mucosal layer or synthetic mucus mimetic drugs on the other [7]. But many adverse effects have been recorded with the drugs that may be reached to cancer development [8]. So numerous studies have directed on the development of another medications depend on on the vast natural capacity of bioactive chemicals found in plants, which are demonstrably less aggressive than traditional pharmaceuticals [9].

Nanotechnology has shown to be very useful in enhancing wound healing therapies. The Nano-Meter scale paved the door for the development of innovative materials for application in cutting-edge medical technologies, as well as the resuscitation of multifunctional Nano carriers’ targeting efficiency. Small molecules of medications might be incorporated into (NPs) or layers to alter their safety, bioavailability, and efficiency. The Nano carrier size has a significant impact on medication pharmacokinetics and pharmacodynamics [10]. The most common cause of a mucosal unhealed lesion is a bacterial infection that leads to gastrointestinal ulcer (GIU), which is caused mostly by Helicobacter pylori is bacteria in adults and children across the world. Because *H.pyloriis* resides underneath the stomach mucous membrane, which supports the gastric epithelium, therapy with appropriate antibiotic concentrations fails. Because of their small size, NPs have shown to be effective in treating gastrointestinal infections because they adhere well to the gastric mucosa and fight bacteria that live there.

Natural medicine is becoming the primary treatment option for all ailments they exert less side effects and capability to combat antibiotic resistance. Propolis’ anti-inflammatory antioxidant, antibacterial,antifungal, antiviral,, and antiseptic,capabilities have made it popular in traditional medicine [11]. It contains varities of many active priciples such as flavonoids, polyphenols, terpenoids, amino acids and steroids [12].

KRG (Korean red ginseng) is a ginseng that has been farmed and matured for at least 4–6 years and has through a thorough cleaning, steaming, and drying procedure [13]. KRG has the most strong numerous pharmacological activities among the many Panax ginseng preparations for treating various human disorders, including cardiovascular diseases, rheumatoid arthritis, and diabetes mellitus.

Gastric mucosa integrity and acid secretion, parietal cell inflammation and atrophy, blood flow, and endogenous agents, particularly nitric oxide (NO) and tumor necrosis factor-alpha (TNF-), should all be considered in protective strategies [14]. Amygdalin is a cyanogenic glycoside that has been shown to have anti-inflammatory properties by suppressing COX-2 and increasing the production of inducible nitric oxide synthase (iNOS) [15]. In some tissues, amygdalin was also shown to decrease TNF-expression [15]. In stomach and duodenal ulcers, pro-inflammatory cytokine levels such as TNF- have been found to rise [16]. Moreover amygdalinhas as antitussive, anti-asthmatic, antiatherogenic, anti-cancer, and anti-ulcer properties and fibrosis inhibition/prevention [17].

The present study was devoted to the varify the gastro protective and the healing potential of different natural materials such as propolis and ginseng in normal and Nano forms besides amygdalin or Vit B17 therapy as a preventive therapy against the ethanol-induced gastric ulcer.

## Materials and methods

### Chemicals

Omeprazole was bought from a local pharmacy; absolute ethanol was purchased from VWR Chemicals, Prolabo (France). A local market provided the propolis and ginseng. Ginseng, Propolis powders, and Vitamin B17 capsules (amygdala), containing NPs, were dissolved in 1% DMSO. Vitamin B17 (Vit B17)was acquired as a bitter raw apricot extract in capsule form with a purity of 98.2% and a concentration of 100 mg per capsule. All of the items in the study were taken orally, whether in conventional or Nano form. The medication dosages were manufactured right before the rats were given them.

### Synthesis and characterizations of Nano Propolis and Ginseng

Commercial Propolis or Ginseng was placed into a photon ball milling vessel with a porcelain ball diameter of 1.8 cm, a stain steel vessel diameter of 7.5 cm, and a rotating speed of 200 rpm for 24 h. We synthesis both nanoproplois and nanogienseng then were characterized using different tools like Zeta potential, particle size, A Malvern (Malvern Instruments Ltd) examined (experimentally optimized) the hydrodynamic size and zeta potential using the technique described in our published work [18]. Scanning electron microscopy (SEM) and High Resolution Transmission Electron Microscopy (HRTEM) (Quanta FEG 250).

### Animals

We utilized 48 healthy male albino rats (Rattus norvegicus) weighing between 120 and 150 g. Rats were bought from the Beni-Suef University Faculty of Veterinary Medicine’s Department of Physiology’s lab animal division. The rats were kept in a standard laboratory environment with 223 °C, 60% humidity, and a 12-hour light/dark cycle. The animal handling methods, which included weighing and gavage procedures, were evaluated and approved by the Institutional Animal Care & Use Committee of Cairo University’s Faculty of Veterinary Medicine (Protocol of Animal Rights for Laboratory Experiments) Approval number (01122022616). A standard 12-hour light/dark cycle was employed throughout the experiment. All of the animals were fed a consistent diet and provided 24-hour access to water. For the formation of stomach ulcers and the treatment of stomach ulcers. The research was carried out after the rats had been acclimatized for 7 days.

### Experimental design

The rats were divided equally into 8 groups (6 animal/ group): Group I (control negative normal rats, where animals were administered saline orally all over the experiment for 7 days (0.5ml of D.W.) orally. Group II (control positive) the animals were orally administrated alcohol (99.9%) at a dose (5ml/kg b.wt.) [19], Group III (animals were orally administrated Omeprazole at a dose (20 mg/ kg) [20] as standard drug, Group IV (The animals were orally administrated Vit B17) at a dose (300 mg/ kg) [21], Group V (The animals were orally administrated propolis at a dose (20 mg/ kg) [22], Group VI (The animals were orally administrated propolis nano (20 mg/ kg) [22], Group VII ( The animals were orally administrated Ginseng (20 mg/ kg) [23], Group VIII (The animals were orally administrated ginseng nano (20 mg/ kg) [23].

### Ulcer induction and estimation method

Seven days prior to ulcer induction, the animals were orally administrated the preparations. Followed by fasting in the next day for 24 h, then animals were orally gavaged absolute ethanol alcohol (99.9%) (5 ml/kg B.wt.). 1 h later the last dose of the tested natural materials were administrated to the intoxicated rats. Following another 1 h the animals were subjected to anaesthesia *via* Ketamine xylazine combination ((0.1 ml/100 gm) with cervical dislocation. The abdominal cavity was longitudinal incised for obtaining the stomach, which opened from the greater curvature and rinsed with physiological saline solution followed by flat pinning on card board for observing the gastrointestinal mucosa gross lesions.and counting the number of ulcers *via* an illuminated magnifying lens (10x). The lesions were numbered and measured along the bigger diameter with a clear ruler. Each of the five hemorrhagic areas equals 1 mm in this computation. The ulcer index was calculated by dividing the total length of long ulcers and hemorrhagic patches in each group of rats by the number of animals (mm). The % of protection was calculated according to the method described by [24].

$$\begin{array}{l}The\,proportion\,of\,protection\\= \,\% \,of\,protection\, \times \,(ulcer\,index\,of\,control\,positive\, - \,ulcer\,index\,of\,treated)\\\div \,ulcer\,index\,of\,control\, \times \,100\end{array} 100$$

### Measurement of ulcer index (UI)

Ulcers in the gastric mucosa show as hemorrhagic extended bands of lesions parallel to the long axis of the stomach. The Ulcer Area is calculated by adding all of the lesions regions for each stomach to get an approximation of the gastric mucosa ulcer (UA). With minor modifications, the Ulcer Inhibition Percentage (UI percent) is determined as reported by [25].

$$UI\% \, = \,[(UIcontrolpositive - UItreated) \div UIcontrol]\, \times \,100\%$$

### Samples collection

After the Macroscopical inspectionfor ulcers. The stomach was immediately separated into two halves. One was frozen and preserved at -80 °C for gene expression analysis, while the other was removed and fixed in Bouin’s solution for histological inspection.

### Histopathological examination

For histopathological examination, gastric tissue samples from different groups under investigation were collected, fixed in Bouin’s fluid for 24 h., dehydrated in ascending grades of ethyl alcohol (50: 100%), cleared in xylene, embedded in paraffin wax and sectioned using rotary microtome at 4–6 μm thickness to obtain tissue sections. sections of all groups were stained with Hematoxylin & Eosin (H&E) and examined under light microscopy [26].

### Quantitative assay of the m-RNA levels Endothelin (ET-1), LTB4, and cas-3 genes ***via*** Quantitative Real time-PCR (RT-qPCR))

Quantitative Real-Time Polymerase Chain Reaction and RNA Extraction (qPCR) Following the manufacturer’s directions, the RNA was isolated from a stomach tissue. The Nanodrop spectrophotometer (Thermo Scientific NanoDrop 1000 Nano UV/Vis spectrophotometer) was used to measure the concentration of total RNA at a 260/280 nm ratio [27]. Total RNA (1ug) was used for complementary DNA (cDNA) synthesis using a RevertAid First Strand cDNA Synthesis Kit (Thermo scientific), in accordance with the manufacturer’s instructions. qPCR was carried out using SYBR™ Green PCR Master Mix (Thermoscientific Cat number: 4,309,155). ABI Prism StepOnePlus Real-Time PCR System (Applied Biosystems) according to the manufacturer’s instructions. For standadarization the expression level of the target genes relatively, Beta Actin (actb) was used as the reference gene [28]. Sequences of primers are displayed in Table (1).The data obtained from the qRT- PCR were analyzed using CT, ΔCT, ΔΔCT, and 2- ΔΔCT [29].

**Table 1:** The primer sequences

	Sense	Antisense	Amplicon	Accession no
*Edn1*	GAGCCCTATGGCCAACTCTG	GCGGATGCAAACGAAGACAG	167	NM_012548.2
*Ltb4*	GTAGTCAAGCTGCTCGAGGG	TCACAGGCTTAACCTGGCTT	351	AB025230.1
*Cas-3*	GAGCTTGGAACGCGAAGAAA	TTGCGAGCTGACATTCCAGT	221	NM_012922.2
*Actb*	CCGCGAGTACAACCTTCTTG	CAGTTGGTGACAATGCCGTG	297	NM_031144.3

### ELISA for histamine

The specimens were homogenized in a cold buffer (PBS) for determing the levels of histamine *via* ELISA method, following the manufacturer histamine kit instructions (Sunlong Biotech Co., China).

### Statistical analysis

The information was provided as a mean with standard deviation (S.E.M.). Statistical significance was determined using SPSS (version 20.0) software and Snedecor’s one-way analysis of variance (ANOVA), followed by Tukey’s post-hoc test for multiple comparisons (IBM SPSS Statistic 20.0, Armonk, NY, USA). P values less than 0.05 were considered statistically significant.

## Results

### Characterizations of nano propolis and nano ginseng

**Fig. 1:**
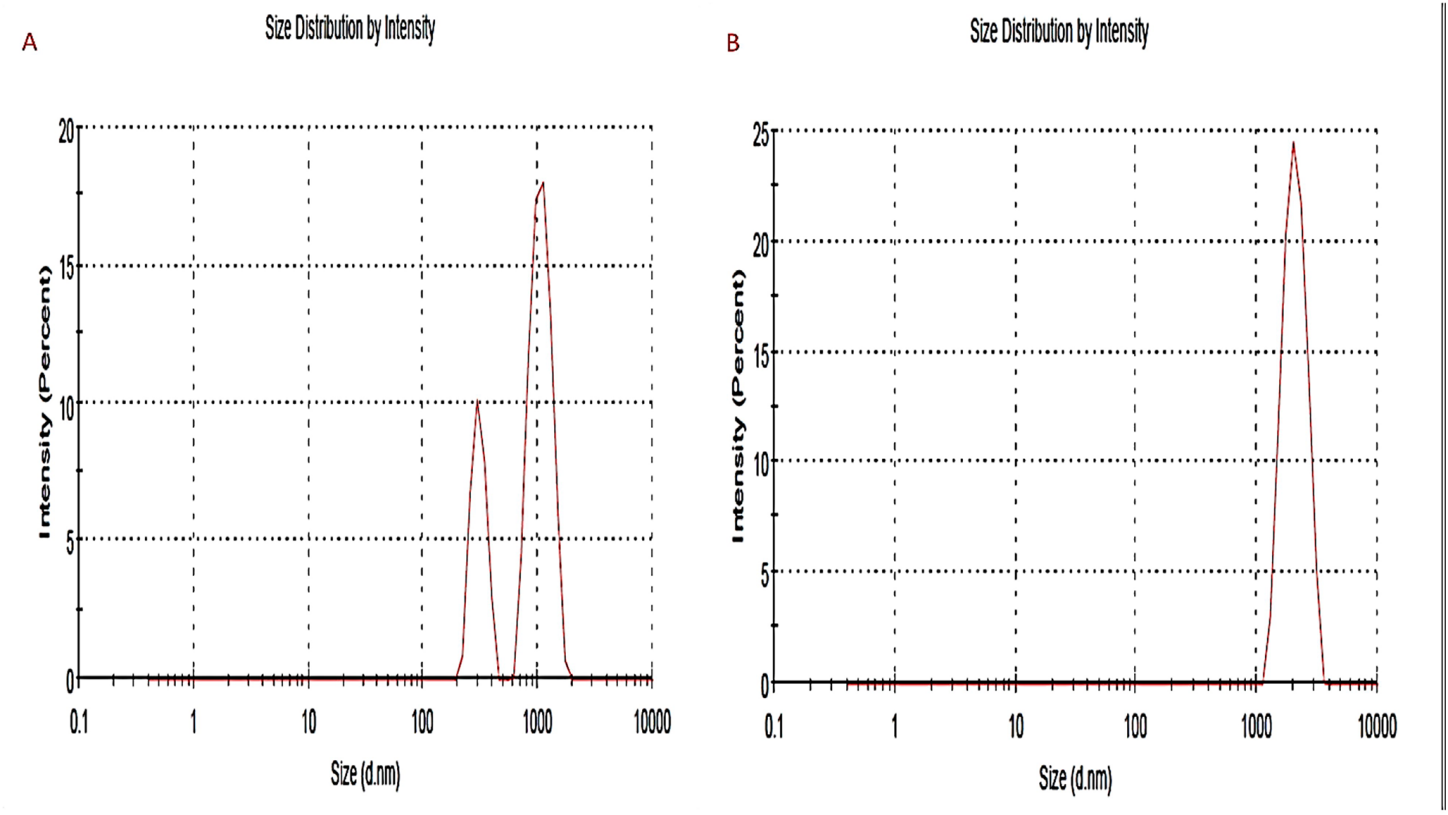
Zeta Sizer of Nano Propolis **(A)** (300–900 nm) and nano Ginseng **(B)** (300 nm)

**Fig. 2:**
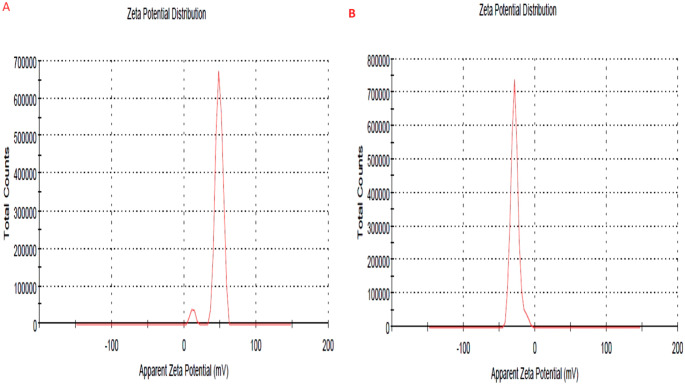
Zeta Potential of Nano Propolis **(A)** (+ 30 mV) and nano Ginseng **(B)** (-28 mV)

**Fig. 3:**
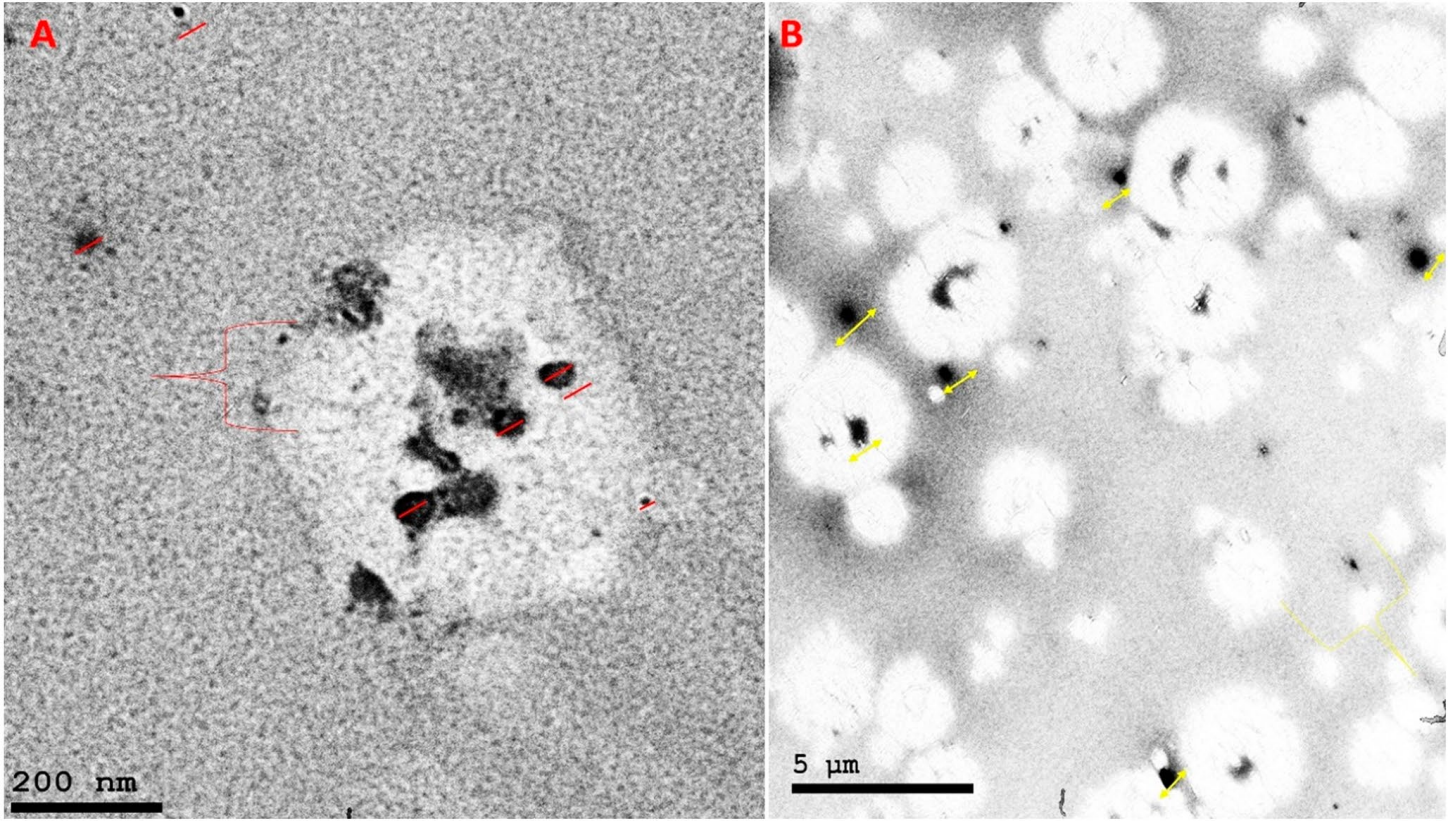
SEM images of nano propolis and ginseng showing the size and the surface morphology of the nanoparticles

**Fig. 4:**
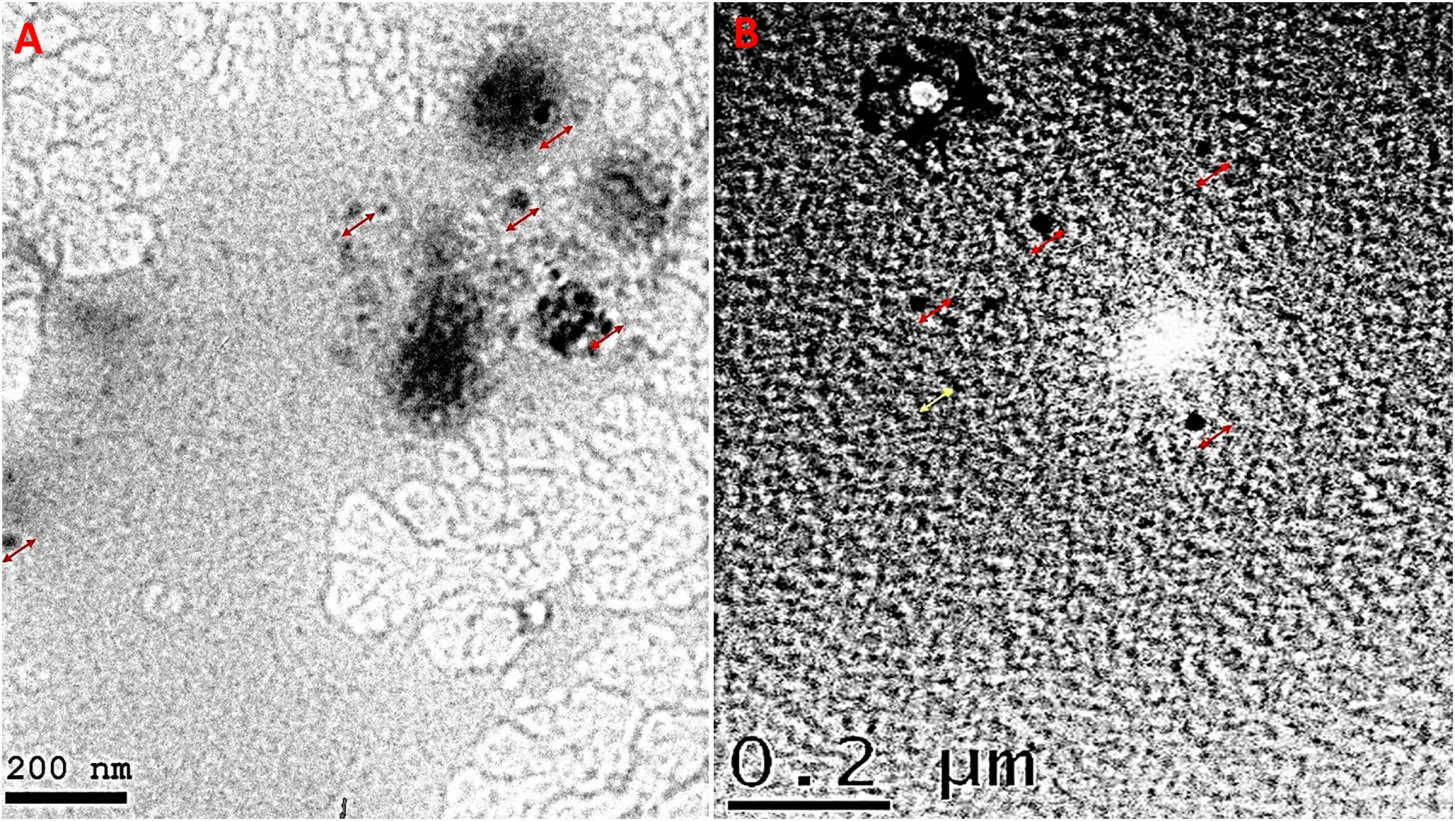
High Resolution Transmission electron microscopy images showing obvious nano propolis **(A)** and ginseng **(B)** without aggregation

As the measurement was repeated more than one, the mean of readings was obtained to calculate the average particle size, the particle size of both propolis and ginseng nanoparticles (NPs) in the present study was approximately 200–300 nm. The size of NPs was also validated by the results of the SEM. The particle size of the NPs was impacted by preparation for both synthetic materials Fig. (1). Our study revealed that Zeta potential values of prepared propolis and ginseng were about + 30: -28 mV Fig. (2), respectively bringing to light high stability and good quality result.SEM revealed that the prepared materials were detached with nearly spherical surface Fig. (3). SEM also gave insights on particle size. The surface morphology of synthesized materials was depicted by SEM. HRTEM analysis was used as a further proof of this nanostructure.(HR-TEM) images of propolis and ginseng NPs revealed that particles werespherical in shape with size ranged between 200 and 500 nm indiameter confirming the mean diameter of the NPs measured Fig. (4).

### Macroscopical examination of induced gastric ulcer

**Fig. 5:**
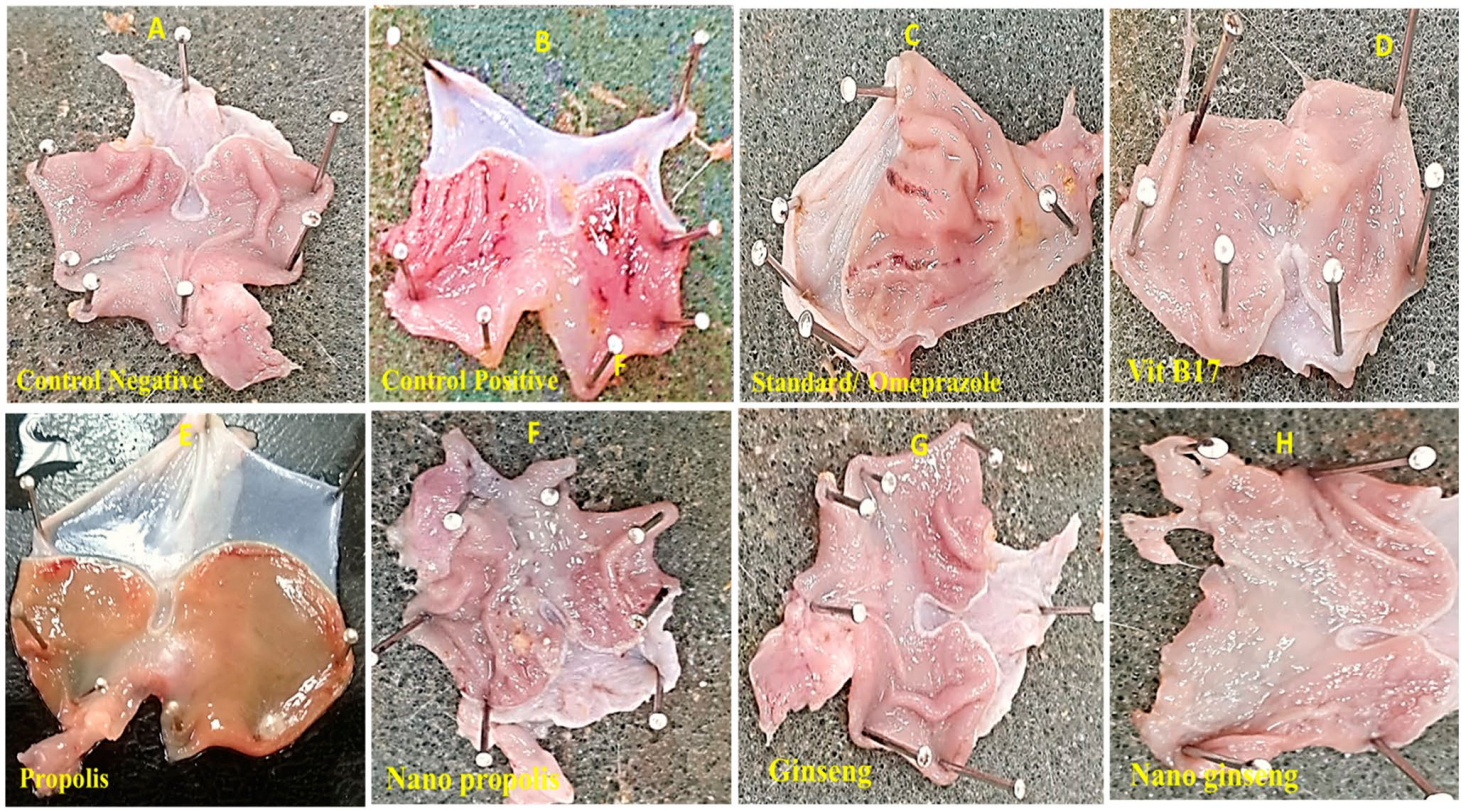
Macroscopical appearance of the induced gastric ulcers in different groups

Propolis, ginseng and Vit b17 pretreatment reduced ethanol-induced damage across the whole stomach mucosa surface with hyperhaemia and longitudinal linear red lesions and. Pretreatment with omeprazole decreased ethanol aggressiveness on the stomach mucosa. Redness, erosions and ulcers were observed locally. Pretreatment of all investigated materials, whether in their normal or nano form, considerably reduces alcohol-induced damage in a dose-dependent way, assuring gastric mucosa protection. Biotechnological drugs are taken orally. The administration of oral administration 99.9% absolute ethanol to 24 h fasting rats resulted in a larger ulcer index in the non-treated control group *(P* < *0*.*00)* in comparison to other treated groups Fig. (5).

### Ulcer number ulcer index and percent of protection estimation

**Fig. 6:**
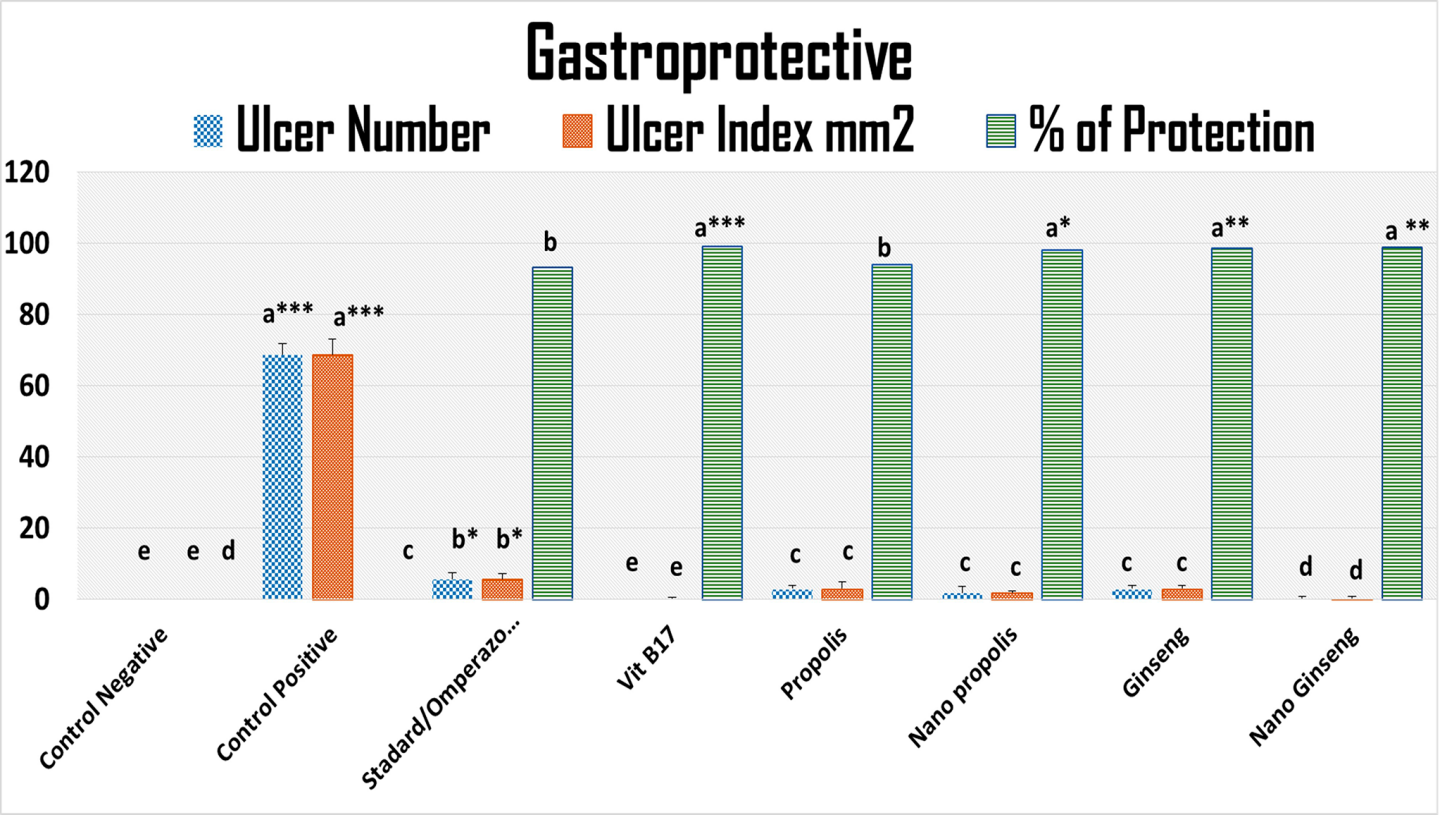
ulcer number, ulcer index, and percent of protection estimation

Significant decrease in the ulcer index and ulcer number (mm^2^) was observed in groups pre-treated with Vit B17, nano ginseng, nano propolis, and normal ginseng (*p* < *0*.*000)*; propolis and standard omeprazole *(p* < *0*.*00)* with higher protection rate when compared to a control non-treated group (positive rats). A Standard group administered with Omeprazole also showed significant decrease (*p* < *0*.*00)* in the ulcer index compared to a control group Fig. (6).

A control non treated rat has showed no ulcers lesions or protection % as appeared in Fig. (6). The percent of protection were significantly (*p* < *0*.*000)* higher in the rats treated with Vit B17, nano and normal ginseng, nano standard and normal propolis when compared to control negative and positive rats (Fig. 6 ).

### Macroscopical examination of gastric gases

**Fig. 7:**
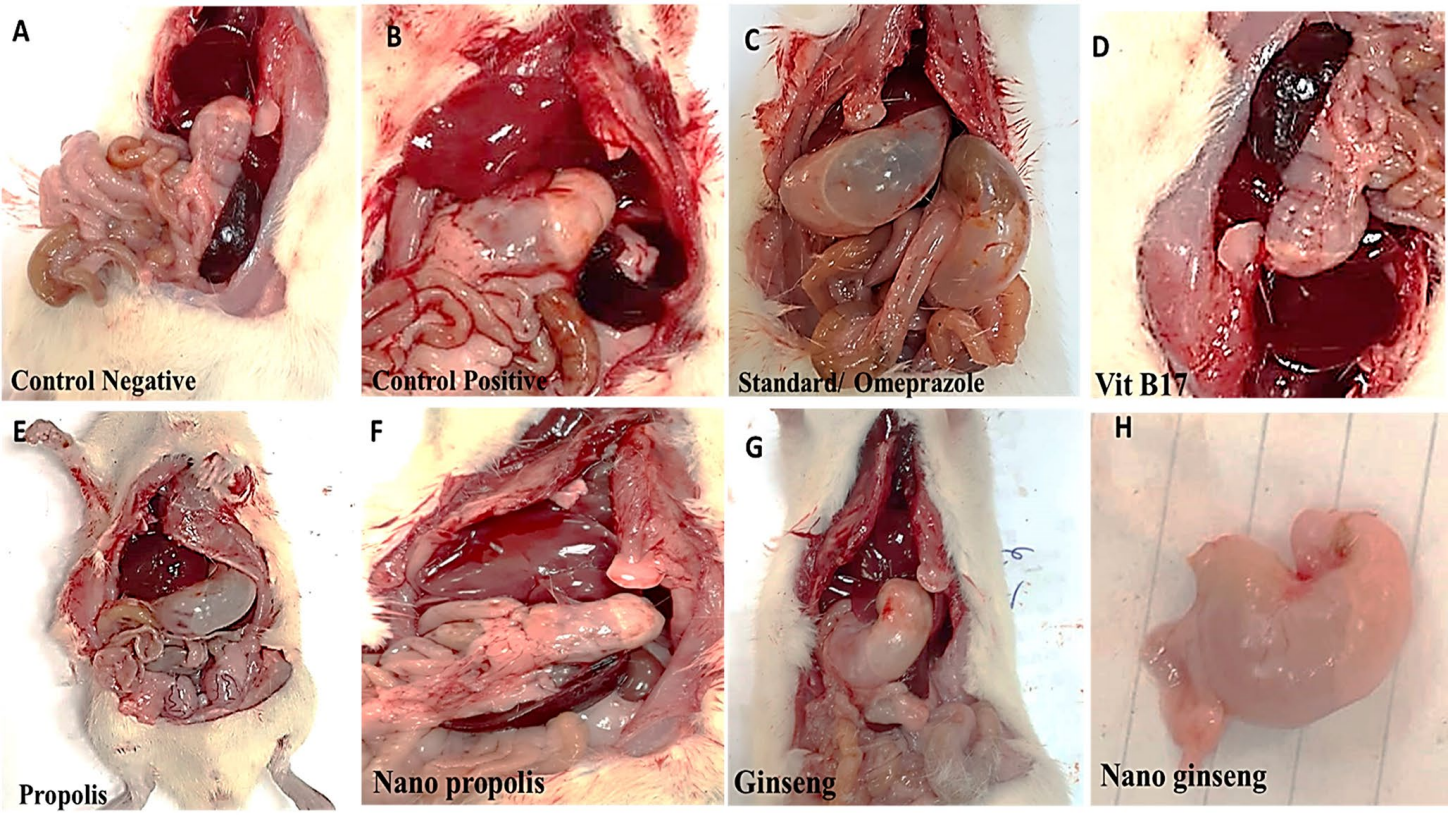
Ulcer number, ulcer index, and percent of protection estimation

The production of gastric ulcer in the rat by a necrotizing substance such as ethanol resulted in mucosal damage characterized by submucosal edema, increased secretory products of the cell, and alterations in microcirculation. The development of gastric ulcer lesions in rats was prevented by pretreatment Vit B17, nano-ginseng, normal ginseng, nano propolis and propolis and omeprazole respectively. In the control positive non-treated group, ulcer intensity is detected by ulcer area, as seen macroscopically Fig. (7). Also gases were observed in the different treatments as the gases levels intra gastric were clearly declined in Vit B17 and nano propolis treated rats as that’s of control negative non-treated normal rats. While the gases severely stretched the gastric dimensions in omeprazole, nano ginseng, ginseng and normal propolis respectively in the degrees of gases distension Fig. (7).

### Histopathological examination

**Fig. 8:**
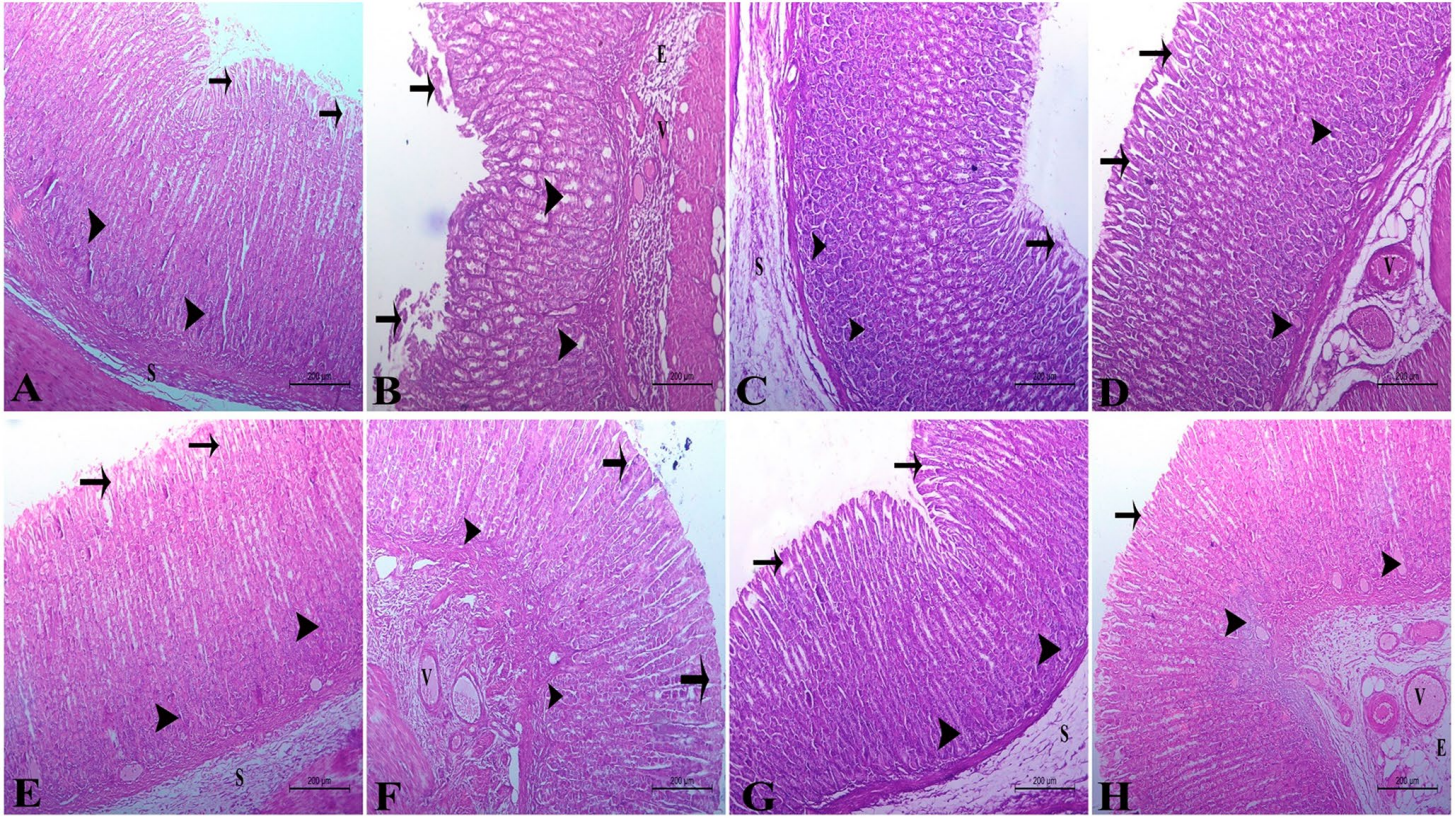
Photomicrograph of an adult male albino rats’ gastric tissue sections showing; **(A)** Control negative group possessed a normal architecture of the gastric tissue with normal surface epithelium (arrows), normal gastric glands (arrowheads) and normal submucosal layer (S). **(B)** Control positive group appeared severely alliterated with degenerated, destructed and sloughed surface epithelium (arrows), degenerated gastric gland (arrowheads). The gastric submucosa showed sever congestion and dilatation of blood vessels (v) as well as marked edema (E) around these vessels. **(C)** Omeprazole treated group showed normal surface epithelium (arrow), normal gastric glands (arrowheads) as well as normal submucosal layer (S). **(D)** Vit B17 treated group showed mild degenerative changes in surface epithelium (arrow) and gastric glands (arrowheads) as well as congestion (V) and edema (E) in the submucosal blood vessels. H&E stain,scale bar: 200 μm. **E)**Propolis treated group showed normal surface epithelium (arrows) and gastric glands (arrowheads) as well as mild congestion in the submucosal blood vessels (V). **F)** Nano propolis treated group showed normal surface epithelium (arrows), normal gastric glands (arrowheads) as well as normal gastric submucosal layer (S). **G)** Ginseng treated group showed mild degenerative changes in surface epithelium (arrows) and gastric glands (arrowheads) as well as mild congestion in the submucosal blood vessels (V). **H)** Nano ginseng treated group showed normal surface epithelium (arrows), normal gastric glands (arrowhead) as well as normal submucosal layer of the stomach (S)

The light microscopic examination of rats’ gastric tissue sections of control negative group showed a normal architecture of the gastric tissue with normal surface epithelium, gastric glands and normal gastric submucosal layer (Fig. 8A).While the rats’ gastric tissue sections of control positive group showed sever histopathological alternations in the form of degenerated, destructed and sloughed gastric surface epithelium, sever degeneration of gastric gland. The gastric submucosa showed sever congestion and dilatation of blood vessels as well as marked edema around these vessels (Fig. 8B). Regarding the treatment with omeprazole, the examination of rats’ gastric tissue sections of Omeprazole treated group showed normal gastric surface epithelium, normal gastric glands as well as normal gastric submucosal layer (Fig. 8C).In Vit B17 treated group, the light microscopic examination of rats’ gastric tissue sections of Vit B17 was showed mild degenerative changes of the gastric epithelium and glands as well as congestion and edema in the gastric submucosal blood vessels (Fig. 8D). For propolis treated group the rats’ gastric tissue sections were showed normal histological architecture of gastric surface epithelium and gastric glands, while the gastric submucosal layer showed a mild congestion of the blood vessels (Fig. 8E). On the other hand, nano propolis treated group was showed normal histological picture of gastric surface epithelium, glands and submucosal layer (Fig. 8F). For ginseng treated group, there are mild degenerative changes of the gastric surface epithelium and gastric glands with mild congestion of the gastric submucosal blood vessels (Fig. 8G). In contrast the nano ginseng treated group was showed normal histological picture of gastric surface epithelium and gastric glands as well as normal gastric msubmucosal layer (Fig. 8H).

### mRNA expression rate of ET-1, LTB4, and cas-3genes

**Fig. 9:**
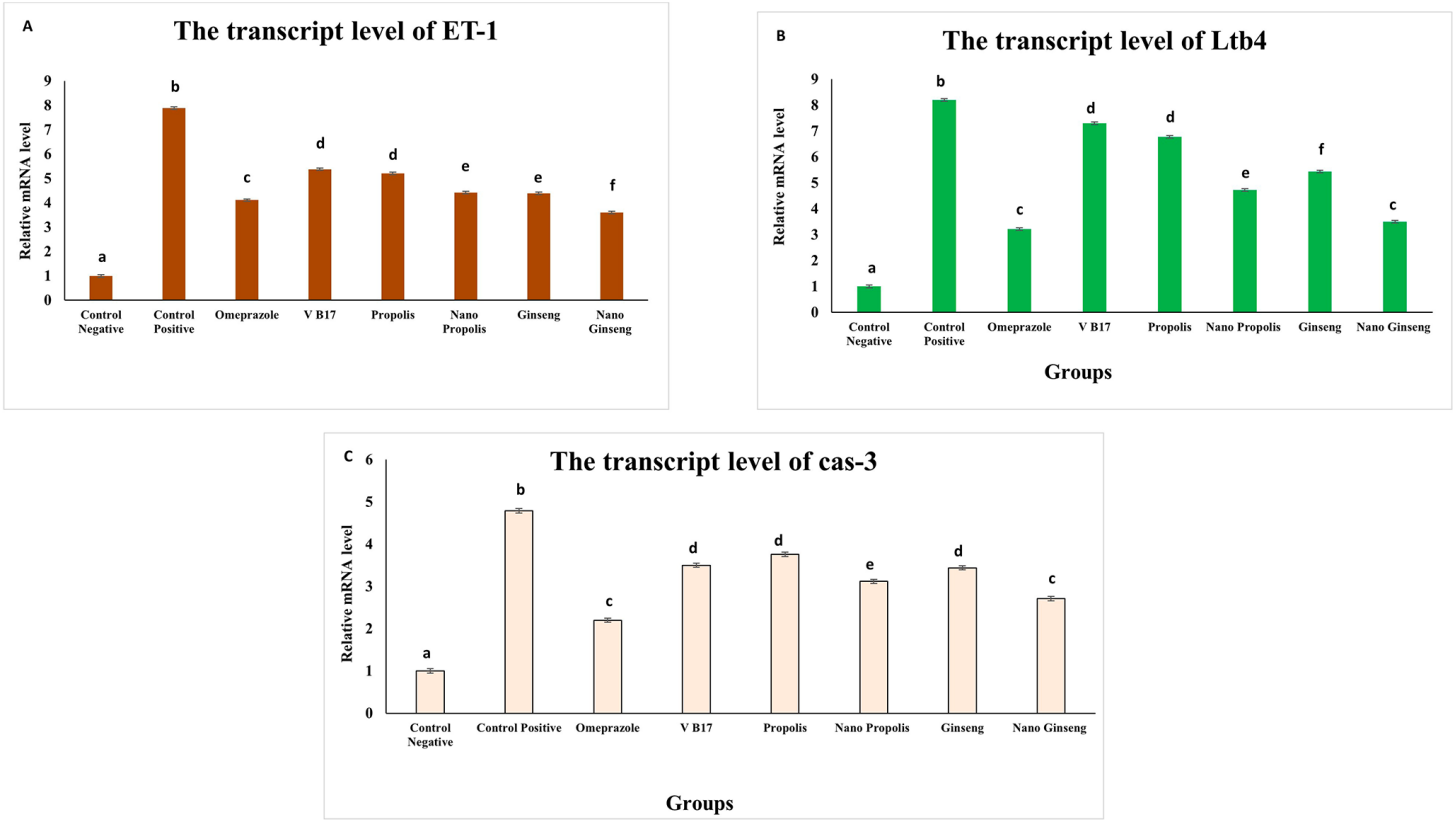
The transcript level of **(a)** ET-1; **(b)** Ltb4; **(c)** cas-3 genes in different groups. Values are presented as mean ± SEM. (n = 6 rats/group). Different superscript letters indicate statistically significant difference at p < 0.05

The ET-1, Ltb4 and cas-3 mRNA showed significant up-regulations in the control positive group compared to the negative control one. The vitamin B17, propolis, nano propolis, ginseng and nano ginseng co-treatments significantly modulated the expression levels of these genes if compared to both the positive and negative control groups. However, the nano ginseng group showed the maximum protective effect compared to the treated groups (Fig. 9).

### ELISA for histamine

**Fig. 10:**
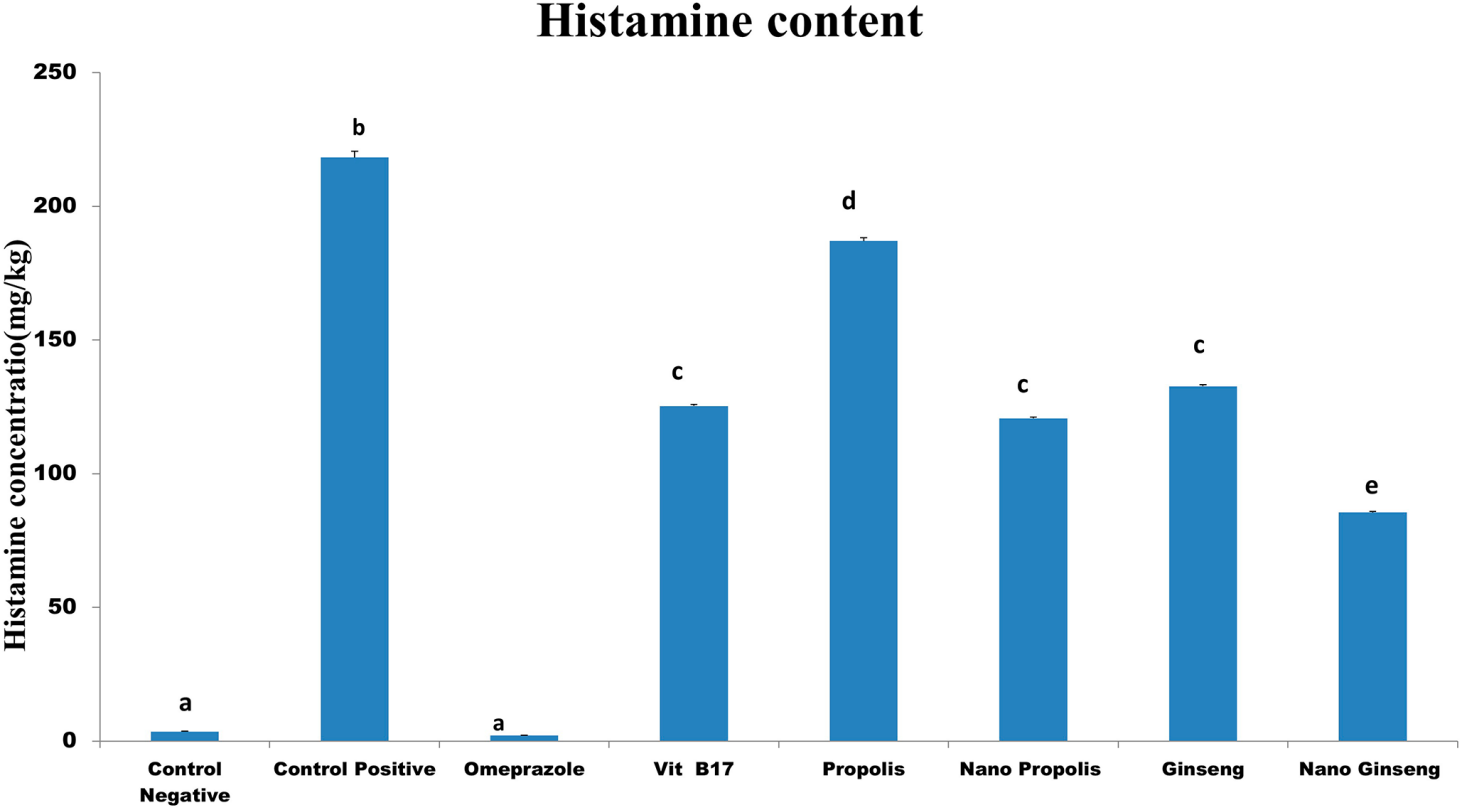
Histamine content in the different groups. Histamine contents in the treated groups. Values are represented as mean ± SEM (n = 3). Different small letters mean a significant difference between groups at P ≤ 0.05

Figure (10) was illustrated that the histamine content was significantly elevated in ethanol intoxicated rats in comparison to control negative and the orally administrated plant extracts groups. Furthermore, the concentration was markedly increased in the treated groups in comparison to control negative and omeprazole groups.

## Discussion

Alternative medicine research focuses on developing novel treatment methods that are effective in preventing stomach injuries or ulcerations from forming, as well as healing them with low or no adverse effects if they have already happened. The current study compared two allopathic reference drugs, Omeprazole and Ginseng I normal and nano form, to see how effective natural antioxidant and anti-inflammatory materials (Vit B17, propolis, and Ginseng I normal and nano form) were at preventing and healing acute gastric ulcers caused by absolute ethanol.

Our data have shown that pre-treatment with different natural materials causing great protection against the potent ulcerative effect of absolute ethanol, that representing in reduction the severe ulcers, lesions, haemorrahges, and necrosis in of the mucosal wall. The great protective efficacy was recorded with Vit B17, nano and normal ginseng, nano and normal propolis. Regarding protection with Omeprazole, variable results were obtained, it has the ability to decrease the mucosal lesions and ulcers, while the potent ethanol ulcerogenic effect still with severe gases formations. The current findings are in agree with the previous reports about the mucosal ulcerative effects of ethanol [30]. Ethanol was noted to cause severe microvascular alterations, alterate the integrity of gastric mucosa, exfoliation of the cellular epithlium, inflammation, haemorrahage, and friability. The toxic effects of ethanol were originally linked to the formation of an abnormally high level of oxidative stress in the stomach mucosa, which resulted in increased generation of reactive oxygen species (ROS). It has been established that alcohol use causes a reduction in mitochondrial membrane potential, resulting in a disruption in mitochondrial electron chain transport and an excess of the oxygen free radical O^2 −^ [19]. It’s been shown that ethanol-induced stomach injuries are linked to increased MDA production in the gastric mucosa [31].

At this study both normal and nano propolis had showed good gastro protective activity against experimentally induced ulcers in rats. These findings are similar to that recoreded with [32]. The flavonoid concentration in propolis is generally linked to these biological activities, and these chemicals have pharmacological relevance since they can inhibit stomach ulcer development through antioxidant and antisecretory processes [33]. Kaempferol phenolic substances extracted from propolis suppressed the pro-inflammatory response in injured stomach wall, boosted production of (nitric oxide) NO, and conserved the glycoprotein of gastric mucus [34]. By stimulating PGE2 in gastric epithelial cells, flavonoids present in propolis can stimulate and enhance mucus and bicarbonate production, as well as impact proton pump activities in stomach parietal cells [35].

Propolis is recognized for its antioxidant properties; various studies have shown that it can reduce oxidative stress in animals that cause stomach ulcers [36]. Propolis’ antioxidant properties are apparently implicated in its gastro protection in NSAID-induced gastric ulcers [37].

Flavonoids found in both propolis and ginseng have been shown to have antisecretory and cytoprotective properties in the models of gastric injury [38].

In terms of ginseng efficacy, it outperformed propolis in both regular and nano form in terms of protective activities. Ginseng increased baseline stomach mucosal blood flow in intact mice in a prior research. Using the hydrogen gas clearance technique, it has also been established that ginseng enhances systemic blood flow in rats, including the liver, spleen, kidney, and stomach mucosa [39]. As a result of the vaso-relaxing activity of KRG due to saponin in the stomach mucosal artery in the resting state, our findings imply that KRG enhanced gastric mucosal blood flow. The medicine, on the other hand, has the potential to cause constipation and diarrhea as a side effect. In this study, Vit B17, was found to have strong gatroprotective action against ethanol-induced stomach ulcers in rats. According to a recent study, amygdalin administration was beneficial for alcohol-induced stomach ulcers, and gastric mucosa protection may be mediated through TNF-suppression and gastric mucosal NO generation, it’s also used to treat and prevent migraines, high blood pressure, and other inflammatory conditions [40].

Our findings imply that amygdalin’s anti-inflammatory properties are due to transcriptional mRNA reduction of pro-inflammatory cytokines such as TNF- [41]. TNF- is also capable of suppressing stomach acid production and inducing apoptosis in parietal cells *via* NF-kB expression, according to research [42]. This pathway might play a role in stomach mucosal atrophy and ulcer formation. As a result, suppressing TNF- in gastric tissue and then administering amygdalin can aid in the repair of gastric lesions [43].

Clarification the roles of the Vit B17, Propolis, nano propolis, ginseng and nano ginseng in modulating gastric ulcer, histamine production and gene expression of ET-1, LT-4 and Cas- 3 is the target from the existing study.

Our results showed that all treatments greatly slowed the progression of the gastric ulcer induced by absolute ethanol in rats. By acting as an anti-inflammatory, antioxidant, and mast cell activity regulator, all treatments protect the stomach mucosa from the harm that ethanol causes. Their beneficial effects were shown to minimize the stomach lesions by down regulation of the ET-1, Ltb4 and cas-3 with controlling the histamine generation which favored gastric healing.

In the gastrointestinal tract, a powerful smooth muscle cells contraction and internal gastric mucosal leak may be happened under the histamine effect [44], it may be causing losing of electrolyte, plasma protein, electrolyte and water, resulting in gastric mucosal hemorrhagic destruction specially with high levels of gastric acid [45].

The optimal target for modulation the expression of the pro-inflammatory molecule in gastric ulcers is NF-B [46]. It becomes active in response to stimulation and controls the production and transcription of downstream genes, including LTB4, as well as cytokines that promote inflammation [47]. LTB4 is a crucial inflammatory mediator, a strong selective neutrophil chemotactic agent that can draw in and activate neutrophils, causing a large-scale generation of inflammatory byproducts that intensifies the local inflammatory response [48]. When compared to treated groups, the expressions of LTB4 were lower in the control positive group. The obtained results supported our hypothesis that all of the study’s treatments could have gastroprotective and anti-inflammatory effects.

The used treatments in the present research were down regulated the expression level of Cas-3. That come in agree with the previous findings, thereby cellular viability improvrments and apoptosis reduction [49]. Pathogenesis of the gastic injury is modulated greatly by ET-1. The up-regulated ET-1 levels that associated with inflammation either local or systemic, affect several proinflammatory cytokines secretion such as TNF-alpha [50]. Our recordings of ET-1 and LTB4 up-regulations levels at induction of ulcer then slow down regulation with healing strongly infer a key role for ET-1 in activating the protracted mucosal inflammatory responses that interfere with termination of the apoptotic events essential for the effective repair process.

## Conclusion

The resulted ulcer from acute administration of absolute ethanol could be tolerated through oral aministarion of the natural remedies, in which Vit B17 besides both propolis or ginseng either in normal or nano form, they exerted agreat therapeutic and prophylactic aptitudes, they contain many biocompounds acting in both ways complementary and synergistic effects to protect the gastric layers besides their antioxidant, anti-inflammatory and antibacterial activity. The clinical applications of these natural materials as aremedy for gactric ulcer should be considered. On theother hand there is alimited evidence about the gastro protective activity of tested natural materials currently.

## Data Availability

All data will be available on request.

## List of abbreviations

ASA: Acetylsalicylic acid
Cas-3: caspase-3
ET-1: Enothelin-1
GIU: Gastrointestinal ulcer
GPx: Glutathione peroxidase
KRG: (Korean red ginseng)
LT-4: leukotriene4
NO: Nitric oxide
NSAIDs: Non-steroidal anti-inflammatory medicines
PPIs: Protein pump inhibitors
ROS: Reactive oxygen species
SEM: Scan electron microscopy
TNF-α: Tumor necrosis factor-alpha
UA: Ulcer area
UI: Ulcer Index
XRD: X-ray diffraction
